# Left ventricular stroke volume measurement by impedance cardiography correlates with echocardiography in neonates

**DOI:** 10.1186/cc10832

**Published:** 2012-03-20

**Authors:** ME Blohm, J Hartwich, D Obrecht, G Müller, J Weil, D Singer

**Affiliations:** 1 University Medical Center Hamburg - Eppendorf, Hamburg, Germany; 2 University Center for Cardiology and Cardiothoracic Surgery, Hamburg, Germany

## Introduction

The aim of this study was to validate impedance cardiography (electrical velocimetry (EV)) as a continuous noninvasive cardiac output monitoring in neonates and infants. As the reference method, discontinuous transthoracic echocardiography (TTE) was used.

## Methods

In a prospective single-center observational study, simultaneous left ventricular stroke volume (LVSV) measurements by EV (using an Aesculon^® ^Monitor) and by TTE were compared. LVSV measurement by TTE was based on the aortic valve velocity time integral multiplied by the area of the aortic valve outflow tract. A total of 102 healthy neonates with normal biventricular cardiac morphology (including PDA or patent foramen ovale) were included - further patient details: 43 female, 59 male, median weight 3.32 kg, median length 51 cm, median age 49.24 hours, mean heart rate 133 ± 22/minute. In total 328 simultaneous LVSV measurements in triplicate irrespective of respiratory cycle were analyzed.

## Results

Significant correlations (*P *< 0.05) were noted between EV-LVSV and body weight, TTE-LVSV and body weight, EV-LVSV and age, TTE-LVSV and age. A significant inverse correlation was seen between EV-LVSV and heart rate, and TTE-LVSV and heart rate. No significant correlation was found for EV-LVSV and age (if age ≤120 hours). No significant effect was seen for a small persistent foramen ovale (*n *= 66) and a small PDA (*n *= 26) on EV-LVSV and TTE-LVSV in the observed cohort. Bland-Altman analysis of logarithmic data showed a bias of the EV-LVSV measurements in comparison to the TTE-LVSV measurements with smaller LVSVs resulting in lower EV than TTE measurements and higher LVSVs resulting in higher EV than TTE measurements. The bias defined by the difference of the means of the two methods was 9.65%, with a mean percentage error of the individual measurements of 55%. Based on the Bland-Altman analysis, a deduced approximated correction factor between TTE-LVSV and EV-LVSV was TTE-LVSV = EV-LVSV^0.539 ^× 10^0.335 ^≈ (√EV-LVSV) × 2.2.

## Conclusion

Correlation between EV and TTE in LVSV measurement was significant. Bland-Altman analysis showed that - despite a large mean error of the individual measurements of 55% - the bias between the means of the two methods was only 9.65%. A correction factor between TTE and EV could be deduced.

